# Multivariate Analysis of Associations between Patellofemoral Instability and Gluteal Muscle Contracture: A Radiological Analysis

**DOI:** 10.3390/jpm12020242

**Published:** 2022-02-08

**Authors:** Qihang Su, Yi Zhang, Yuanzhen Zhang, Jie Li, Chao Xue, Hengan Ge, Biao Cheng

**Affiliations:** 1 Department of Orthopedics, Shanghai Tenth People’s Hospital, School of Medicine, Tongji University, No.301 Yanchang Middle Road, Shanghai 200072, China; 15221378017@163.com (Q.S.); equilibrium@tongji.edu.cn (Y.Z.); 1931177@tongji.edu.cn (Y.Z.); dr_xuechaoshiyuan@163.com (C.X.); ghahayeah@163.com (H.G.); 2 Department of Orthopedics, Zhabei Central Hospital, No.619 Zhong Hua Xin Road, Jingan District, Shanghai 200070, China; dr_lijie@163.com

**Keywords:** gluteal muscle contracture, anterior knee pain, patellofemoral instability, arthroscopic GMC release

## Abstract

The purpose of this study was to investigate the associations between gluteal muscle contracture (GMC) severity and patellofemoral instability and evaluate the reliability of novel indicators by multivariate analysis. Clinical and imaging data from 115 patients with GMC were collected for retrospective analysis. Two novel indicators were used to evaluate GMC severity (knee flexion angle and hip flexion angle, feet distance), and two additional novel parameters were used to reflect patellofemoral instability [patellar displacement vector (L, α), patella-femoral trochlear (P-FT) area, and femoral-trochlear-patella (FT-P) area]. In this study, patients with moderate contracture were dominant, and 35.65% also experienced anterior knee pain after physical activity. Ordered logistic regression analysis indicated that a more serious GMC represented a higher risk of lateral tilt and lateral displacement of the patella. Multivariate analysis showed that feet distance was a reliable indicator for evaluating the severity of GMC. The results showed that the more serious the GMC, the more significant the difference between the P-FT area and the FT-P area of the patellofemoral joint space. L, patellar tilt angle, patellar congruency angle, and lateral patellofemoral angle were independent risk factors for this difference. A more serious GMC represents a higher risk of patellar subluxation.

## 1. Introduction

Gluteal muscle contracture (GMC) is a clinical syndrome characterized by limited hip joint function attributed to gluteal muscle and corresponding fascia contracture. Patients typically present with an increased hip abduction angle while flexing the hip and knee, an inability to squat down while closing knees together or cross their legs, a positive Ober’s sign, an audible snapping sound during squatting, and even claudication gait in severe cases [1,2]. GMC is mainly associated with repeated intramuscular injection in the buttocks of patients in childhood, while a few cases are related to trauma and autoimmune function in terms of etiology [3]. As reported previously, the incidence of GMC in China ranges from 1–2.5% in China [4]. As is well-known nationwide, the diagnosis and treatment of GMC are parts of the characteristic specialties of our group-affiliated department, and on average, 2–3 GMC patients visit the hospital every week. In our hospital, such patients are mainly treated with minimally invasive therapy by arthroscopic GMC release [5]. During clinical practice, the vast majority of patients complain of an inability to squat down while closing their knees together or crossing their legs upon arrival. Most of these patients present with knee pain or discomfort; this is aggravated after vigorous activity and can be relieved after minimally invasive GMC release. 

Correlations exist between anterior knee pain and GMC-induced patellofemoral instability. This may trigger repeated patellar dislocation and further develop into patellar cartilage softening or patellofemoral osteoarthritis. As previously reported, patellofemoral instability can be efficiently ameliorated by means of arthroscopic GMC release, thus alleviating the knee pain [6]. A study conducted by Zhao et al. [7] analyzed the diagnosis and treatment of 36 GMC patients with anterior knee pain and confirmed that arthroscopic GMC release can relieve anterior knee pain, thus contributing to the prevention of patellofemoral arthritis. Another study reported by Wang et al. [8] on the diagnosis and treatment of 52 GMC patients with knee osteoarthritis revealed that GMC can affect knee joint stability and induce premature degenerative changes in the knee joints, thus leading to the occurrence of knee osteoarthritis. In addition, Jia et al. [9] reported that compared with arthroscopic GMC release alone, the combination of arthroscopic GMC release and medial patellar retinaculum plication and lateral patellar retinaculum release can better alleviate anterior knee pain resulting from long-term GMC and reduce patellar lateral displacement, thus indicating that the preoperative assessment and selection of surgical approaches are of great significance for patients with different conditions of GMC and anterior knee pain. However, most existing studies have only reported single-factor analysis for the correlation between GMC and patellofemoral instability. Unfortunately, the influence of multiple confounding factors such as GMC severity and congenital patellofemoral dysplasia has not been considered. This vital omission is not conducive to preoperatively evaluating and formulating clinical therapeutic strategies for GMC with patellofemoral instability or for predicting and assessing the postoperative improvement rate of patellofemoral instability.

**Figure 1:**
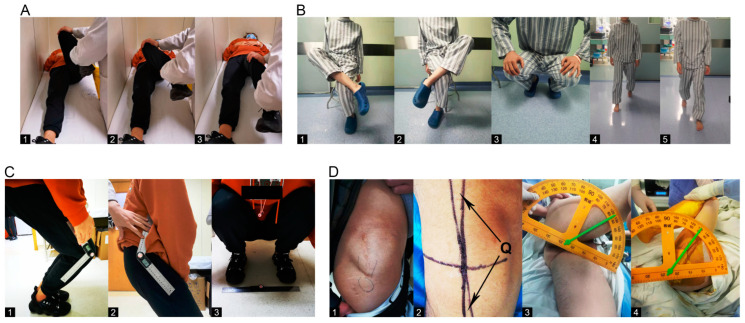
Physical examination of GMC patients. (**A**) In addition to the Ober’s sign, passive induction of hip snapping and hip muscle tightness checks are the most commonly used physical examinations in the clinic. Specifically, the patients lie in a supine or lateral position and (**1**) passively flex the hips and knees, (**2**) adduct the hip joint and abduct the knee joint, and then (**3**) gradually straighten the lower limbs. For patients with severe GMC, obvious snapping can be induced, and the sense of tightness can be felt in the hip. (**B**) (**1**,**2**) This patient tested positive for the cross-leg test and was unable to cross his legs; (**3**) The patient found it difficult to squat with both knees together, producing frog-like legs; (**4**,**5**) Claudication gait, toe-out gait. (**C**) (**1**,**2**) Angle of hip flexion and knee flexion when the patient squatted maximally; (**3**) Minimum foot distance (Feet distance) required when the patient squatted maximally. (**D**) (**1**) Obvious hip contracture band; (**2**) Quadriceps angle; (**3**) Hip abduction angle in the case of 90° hip flexion and knee flexion under anesthesia; (**4**) Maximum angle of hip flexion under anesthesia.

The diagnosis, severity assessment, and classification of GMC are mainly based on clinical symptoms, physical examination, imaging data, and intraoperative arthroscopic assessment (Figure 1) [1,2,6]. Patellofemoral instability is mainly evaluated by means of physical examination and imaging methods. In addition to routine indicators, four novel assessment indicators were specifically designed for this study. First, the knee flexion angle and hip flexion angle in the case of maximally squatting while closing the knees together (Figure 1(C1,2)). Second, the minimum distance between the feet required for the patient to squat down completely (Figure 1(C3)). Third, in the axial computed tomography (CT) image of the knee joint, a connecting line was made between the highest point of the lateral femoral condyle and the outermost point of the patella on the plane connecting each of the highest points of the medial and lateral femoral condyles; the length of the connecting line was considered as L, and the angle between the plane and the connecting line was regarded as α. Fourth, in the axial CT image of the knee joint, the cross-sectional area between the femur and the patella was measured on the plane connecting each of the highest points of the medial and lateral femoral condyles. This study aimed to investigate the correlations between GMC severity and patellofemoral instability and to evaluate the reliability of novel indicators by performing multivariate analysis with multiple indicators. We also aimed to investigate the efficacy of arthroscopic GMC release. The present study can provide a reliable basis to investigate the correlations between GMC and patellofemoral instability, and offer theoretical references for preoperative assessment and clinical decision-making for GMC patients with patellofemoral instability.

## 2. Materials and Methods

### 2.1. Patient Cohort

In this study, we retrospectively collated data from patients with GMC who were treated by arthroscopic GMC release between January 2018 and December 2020. The inclusion criteria were as follows: (a) patients diagnosed with GMC and treated by arthroscopic GMC release (bilateral) [6]; (b) those aged 18–60 years old; (c) those with complete medical history data relating to physical examination and surgery, and the classification of GMC according to Ye et al. [1] and Zhao et al. [2]; and (d) those who underwent CT examination of the knee joint preoperatively. The exclusion criteria involved: (a) patients who visited the hospital due to a poor curative effect of surgery for GMC in the past; (b) those with an incomplete medical history or imaging data; (c) those with deformity of the pelvis or both lower limbs (such as an asymmetric pelvis or serious unequal length in both lower limbs); (d) those with a history of severe trauma or surgery of the pelvis or both lower limbs (such as pelvic fracture, femoral fracture, patellar fracture, meniscus injury, or cruciate ligament injury), or (e) those with neurological or mental disorders and an inability to cooperate with physical examination.

All operations were performed by the corresponding author. All evaluations and measurements were completed by an experienced clinician and the first author. The Hospital Medical Ethics Committee approved the study protocol, which met the relevant guidelines and regulations of the University Medical Ethics Committee.

### 2.2. Classification of GMC

In this study, GMC was classified according to Ye et al. [1] and Zhao et al. [2], who both put forward a reference system to comprehensively evaluate GMC patients and perform classification. The classification method described by Ye et al. [1] is a common and internationally well-recognized criterion; therefore, this method was employed for grouping analysis in this study. Meanwhile, Zhao et al.’s classification mainly provided references for evaluating the severity of GMC [2].

GMC was classified according to research by Ye et al. [1] as follows. Type A was defined as when contracture occurred only in the iliotibial tract, with the following symptoms: able to squat down while closing knees together but an audible snapping sound accompanied by the gliding of a band-shaped substance during the process of squatting. Type A can be divided into two subtypes: A1 and A2. Type A1 was defined as when the contracture was slight and the lower extremities could be crossed and overlapped (i.e., the patient was able to cross their legs) after hearing a snapping sound while flexing the hip. Type A2 was defined as when the contracture was severe and the lower extremities could not be crossed and overlapped (i.e., the patient was unable to cross legs) but hearing a snapping sound while flexing the hip. Type B was defined as contracture occurring in at least two of the tissues involving the iliotibial tract, anterior fibers, and tendon plate as well as the superficial and deep fascia of the gluteus maximus, with the following symptoms: unable to squat down while closing knees together but able to squat down while separating the knees, keeping the knees together after squatting, and an audible snapping sound and gliding of a band-shaped substance during the process of squatting. Type B was classified into three subtypes: B1, B2, and B3. Type B1 was defined as contracture occurring in the iliotibial tract and the anterior fibers and tendon plate of the gluteus maximus concurrently; the abduction angle required for hip flexion was less than 10°. Type B2 contracture occurred in the superficial fascia concurrently, and the abduction angle required for hip flexion was 10–30°. Type B3 contracture occurred in the deep fascia of the gluteus maximus concurrently, and the abduction angle required for hip flexion was greater than 30°. In Type C, in addition to the iliotibial tract, anterior fibers, and tendon plate as well as the superficial and deep fascia of gluteus maximus, contracture occurred in deep tissues such as the gluteus medius, gluteus minimus, the middle fibers of the gluteus maximus, the partial fibers of the piriformis, and the upper ligament of the hip joint, with the following symptoms: able to squat down while separating the knees, and unable to keep the knees together after squatting. Type C was divided into two subtypes, C1 and C2. In Type C1 (known as the severe type by physicians), there was no adhesion or slight adhesion could be found between the contracture tissues, a snapping sound could be heard, and the gliding of a band-shaped substance was observed while flexing the hip, and the intraoperative separation was easy. Type C2 (known as the extremely severe type by some physicians) was associated with serious adhesion between the contracture tissues; there was no gliding and snapping sound regardless of hip flexion in any position. Furthermore, late-shaped depression could be observed in the hip, and the intraoperative separation was difficult.

The severity of GMC was classified in line with research by Zhao et al. [2] as mild, moderate, and severe. Mild GMC involves mild abduction and external rotation deformity of lower limbs, an abduction angle < 15° in the state of hip flexion and knee flexion at 90°, or an affected hip adduction angle < 20° in the extension state of the affected limb; Ober’s sign and the frog squatting sign are weakly positive, and claudication gait was not obvious. Moderate GMC involved moderate abduction and external rotation deformity of the lower limbs (15–60°) in the state of hip flexion and knee flexion at 90°, or an affected hip adduction angle < 10° in the extension state of the affected limb; Ober’s sign and the frog squatting sign was positive, and claudication gait was obvious. Severe GMC involves severe abduction and external rotation deformity of the lower limbs, an abduction angle > 60° in the state of hip flexion and knee flexion at 90°, or an adduction angle < 0° in the extension state of the affected limb; Ober’s sign and the frog squatting sign were strongly positive, and claudication gait was severe.

### 2.3. Measurement of Clinical Parameters

A number of clinical parameters were measured including quadriceps angle (Q-angle) and the three indicators designed by our group for the squatting test.

Q-angle represents the supplementary angle of the angle between the line connecting the anterior superior iliac spine and the midpoint of the patella and the line connecting the tibial tubercle and the midpoint of the patella [10] (Figure 1(D2)). GMC patients are usually unable to squat down while closing their knees together, and a certain distance between the feet was required for the patient to squat down completely. Therefore, two novel indicators were designed by our group for the squatting test including the knee flexion angle and the hip flexion angle in the case of maximal squatting while closing the knees together (Figure 1(C1,2)). We also measured the minimum distance required between the feet in order for the patient to squat down completely (feet distance, Figure 1(C3)).

### 2.4. Measurement of Imaging Parameters for the Patellofemoral Joint

Considering the patient’s willingness, the demand for rapid rehabilitation and discharge, and the impact of CT radiation and knee CT examination was only performed preoperatively in this study. This was followed by the measurement of relevant parameters related to the patellofemoral joint as well as the assessment, analysis, and comparison with normal values for the parameters, as reported in the literature. 

**Figure 2:**
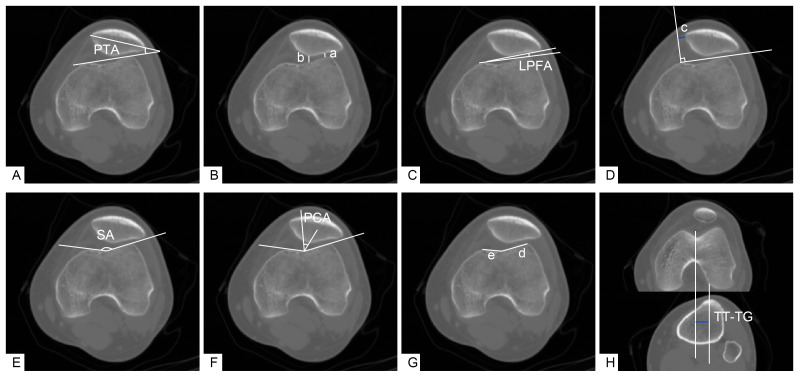
Measurement of assessment indices for patellofemoral instability in knee CT. (**A**) Patellar tilt angle (PTA): the angle between the line connecting each of the highest points of the medial and lateral femoral condyles and the transverse axis of the patella. (**B**) Patellofemoral index (PFI): the ratio of the narrowest width of the medial patellofemoral joint space (b) to the narrowest width of the lateral patellofemoral joint space (a). (**C**) Lateral patellofemoral angle (LPFA): the angle between the tangent line of the lateral articular surface of the patella and a line connecting each of the highest points of the medial and lateral femoral condyles. (**D**) Lateral patellar displacement (LPD): the length between a vertical line connecting each of the highest points of the medial and lateral femoral condyles from the highest point of the medial femoral condyle (c) and the inner edge of the patella. (**E**) Sulcus angle (SA): the angle between the line connecting the lowest point of the femoral trochlear groove and each of the highest points of the medial and lateral trochlear articular surfaces. (**F**) Patellar congruency angle (PCA): the angle between a line connecting the lowest point of the femoral trochlear sulcus and the lowest point of the patellar crest and the bisector of the SA made on the axial image of the patella [14]. (**G**) Sulcus lateral facet ratio (SLFR): the ratio of the lateral articular surface of the femoral trochlear joint (d) to the medial articular surface of the femoral trochlear joint (e). (**H**) Tibial tubercle-trochlear groove distance (TT-TG): the mediolateral distance between the highest point of the tibial tuberosity to the deepest point of the trochlear groove in line with the posterior condylar axis.

Imaging indicators for the routine evaluation of patellofemoral instability (axial CT scan) (Figure 2) were as follows. First, patellar tilt angle (PTA): the angle between the line connecting each of the highest points of the medial and lateral femoral condyles and the transverse axis of the patella [11]. Second, patellofemoral index (PFI): the ratio of the narrowest width of the medial patellofemoral joint space (b) to the narrowest width of the lateral patellofemoral joint space (a); the normal value is <1.6 generally when evaluating the degree of patellar tilt and subluxation [6]. Third, lateral patellofemoral angle (LPFA): the angle between the tangent line of the lateral articular surface of the patella and the line connecting each of the highest points of the medial and lateral femoral condyles. Under normal conditions, the opening of this angle should be outward (set as a positive number). If the opening was inward (set as a negative number) or the two lines were parallel, then this revealed the occurrence of lateral patellar tilt [12]. Fourth, lateral patellar displacement (LPD): the length between the vertical line of the line connecting each highest point of the medial and lateral femoral condyles from the highest point of the medial femoral condyle (c) and the inner edge of the patella. The inner edge of a normal patella should be close to the vertical line, on or beyond the vertical line (set as a negative number); significant deviation from the vertical line indicated the lateral displacement of the patella (set as a positive number) [12]. Fifth, sulcus angle (SA): the angle between the line connecting the lowest point of the femoral trochlear groove and each of the highest points of the medial and lateral trochlear articular surfaces. An angle > 145° was considered as femoral trochlear dysplasia (FTD) [13]. Sixth, patellar congruency angle (PCA): the angle between the line connecting the lowest point of the femoral trochlear sulcus and the lowest point of the patellar crest and the bisector of the SA made on the axial image of the patella [14]. Seventh, sulcus lateral facet ratio (SLFR): the ratio of the lateral articular surface of the femoral trochlear joint (d) to the medial articular surface of the femoral trochlear joint (e); a value > 2.5 was regarded as FTD [15]. Finally, tibial tubercle-trochlear groove distance (TT-TG): the mediolateral distance between the highest point of the tibial tuberosity to the deepest point of the trochlear groove in line with the posterior condylar axis. A TT-TG > 2 cm served as a sign of a pathological lateral position of the tibial tubercle (a surgical indication of tibial tubercle displacement) [16].

**Figure 3:**
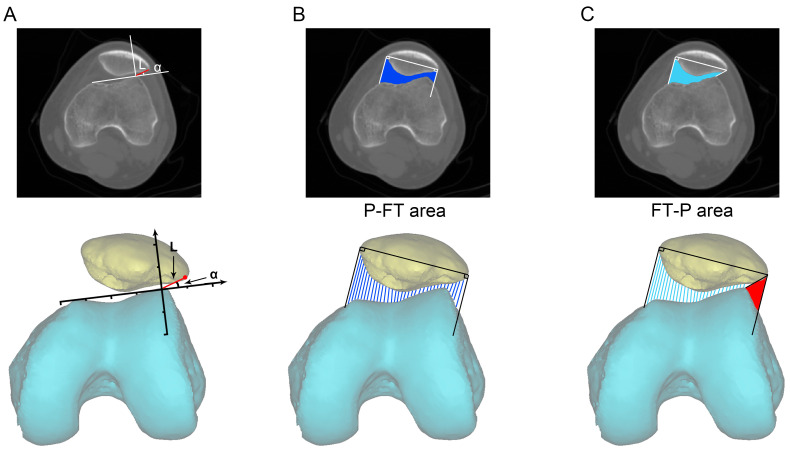
Two newly designed assessment indicators for the patellofemoral joint. (**A**) In an axial CT image of the knee joint, on the plane connecting each of the highest points of the medial and lateral femoral condyles, a connecting line was created between the outermost point of the patella and the highest point of the lateral femoral condyle. The length of the line was considered as L, and the angle between this line and the line connecting each of the highest points of the medial and lateral femoral condyles was regarded as α (with the line connecting each of the highest points of the medial and lateral femoral condyles as the *X*-axis and the line from the highest point of the lateral condyle as the *Y*-axis; this allowed us to establish a coordinate system for L and α). (**B**) Patella-femoral trochlear (P-FT) area: in an axial CT image of the knee joint, the longest axis between the medial and lateral sides of the patella was first drawn on the plane connecting each of the highest points of the medial and lateral femoral condyles, and two lines perpendicular to the long axis were created at the outermost and medial points of the patella. The cross-sectional area from the lower edge of the patella to the femoral trochlear groove between two vertical lines was measured (the area marked in blue). (**C**) Femoral trochlear-patella (FT-P) area: in the axial CT image of the knee joint, the longest axis between the medial and lateral sides of the patella was first drawn on the plane connecting each of the highest points of the medial and lateral femoral condyles. A line perpendicular to the long axis was then drawn at the medial point, and a connecting line was created between the outermost point of the patella and the highest point of the lateral femoral condyle. The cross-sectional area from the lower edge of the patella to the femoral trochlear groove between the two connecting lines was then measured (the area shown in light blue). The area of the red region indicates the difference between the P-FT area and the FT-P area.

In this study, two novel parameters were designed to evaluate the patellofemoral joint, involving L and α as well as the patella-femoral trochlear (P-FT) area and the femoral trochlear-patella (FT-P) area. First, in the axial CT image of the knee joint, on the plane connecting each of the highest points of the medial and lateral femoral condyles, a connecting line was made between the outermost point of the patella and the highest point of the lateral femoral condyle. The length of this line was considered as L, and the angle between this line and the line connecting each of the highest points of the medial and lateral femoral condyles was regarded as α (namely the vector displacement information for the outermost point of the patella and the highest point of the lateral femoral condyle). We assumed that this line could directly reflect the distance and tilt angle for the lateral displacement of the patella (Figure 3A). Second, the patella-femoral trochlear (P-FT) area: in the axial CT image of the knee joint, the longest axis between the medial and lateral sides of the patella was first drawn on the plane connecting each of the highest points of the medial and lateral femoral condyles. Then, two lines perpendicular to the long axis were created at the outermost and medial points of the patella. The cross-sectional area from the lower edge of the patella to the femoral trochlear groove between the two vertical lines was then measured (Figure 3B). We also established the femoral trochlear-patella (FT-P) area: in the axial CT image of the knee joint, the longest axis between the medial and lateral sides of the patella was first drawn on the plane connecting each of the highest points of the medial and lateral femoral condyles. A line perpendicular to the long axis was then drawn at the medial point, and a connecting line was created between the outermost point of the patella and the highest point of the lateral femoral condyle. The cross-sectional area from the lower edge of the patella to the femoral trochlear groove between the two connecting lines was then measured (Figure 3C). It was assumed that the difference in the two areas was increased in the case of lateral displacement of the patella whereas in normal alignment, the patellofemoral joint exhibits a relatively smaller difference between the two areas, or no difference at all (red area, Figure 3C).

### 2.5. Surgical Procedure

General anesthesia was used for all of the arthroscopic procedures. On a normal operating table, the patient was in the lateral decubitus position. The hip was flexed, adducted, and internally rotated to the maximum extent possible without traction. The sterile draping was carried out as usual. Two portals were 3–4 cm apart. An oblique 3 mm incision was created on the skin and subcutaneous tissue for portals. A 4 mm diameter standard 30° scope was inserted at a 30° angle through a proximal portal. The operation space’s fat and fibrous tissues were removed by inserting the digital portal saver. Through the same distal portal as the shaver, a radiofrequency device was inserted. The iliotibial bundles were first sliced in half on both the anterior and posterior sides. Only after the final exploration had been completed was the remaining part of the iliotibial bundle entirely cut, whereas contractures of the gluteus and tensor fascia lata bands were totally cut. After the contractures were eliminated, the leg was progressively moved through the hip’s complete range of motion to ensure there was no clicking. Once the surgeon was satisfied, the fluid was extracted and the skin sutured shut to close the portals. The operation took about 15–20 min in total.

### 2.6. Statistical Analysis

Data were analyzed using IBM SPSS Statistics for Windows, version 20 (IBM Corp., Armonk, New York, NY, USA). The Wilcoxon signed-rank test (non-parametric test) was used to compare related samples including Q-angle, knee flexion angle, hip flexion angle, and feet distance, before and after operation. The correlation and influence between GMC classification (Ye et al. [1]) and other measurement data were analyzed using an ordered logistic regression model. A generalized linear mixed model (GLMM) was used to investigate the correlation and impact of the differences between the P-FT area and FT-P area and other measurement data. For the two models above-mentioned, each variable was subjected to univariate analysis to identify significantly related variables (*p* < 0.10). Then, the screened variables were incorporated into the model for multivariate analysis to identify the final independent risk factors (*p* < 0.05) [17].

## 3. Results

In this study, we enrolled a total of 115 GMC patients with a mean age of 32 ± 6.02 years including 63 females (63/115, 54.78%). Of these, 92.17% (106/115) of patients experienced repeated intramuscular injections during their childhood, 7.83% (9/115) had a history of gluteal muscle trauma in their buttocks, and 35.65% (41/115) of patients suffered from knee pain or discomfort (Table 1). None of the cases involved autoimmune etiology.

According to Zhao et al.’s classification [2], patients with moderate GMC accounted for the majority (n = 59, 51.30%), while those with severe GMC accounted for 36.50%. With regard to Ye et al.’s classification [1], the proportions of GMC patients with type B2, B3, and C1 GMC were 30.40%, 36.50%, and 19.10%, respectively. Patients with these three types of GMC, in accordance with Zhao et al.’s classification [2], could be further categorized into either moderate or severe GMC. In addition, we determined the number of patients with patellofemoral dysplasia based on three indicators (SLFR, SA, and TT-TG). We found that abnormal SLFR, SA, and TT-TG were evident in 6, 23, and 15 cases, respectively. The majority of patients had normal femoral trochlea, which reduced the impact of confounding factors when exploring the correlation between GMC and patellofemoral instability (Table 1).

**Figure 4:**
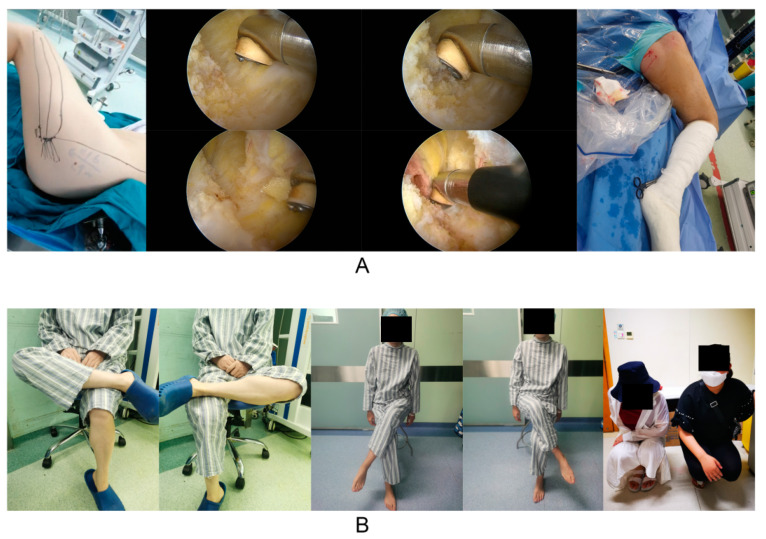
(**A**) During arthroscopic GMC release, severe contractures of the muscles and fascia could be seen under a microscope. (**B**) After surgery, the hip ROM was improved, and the patient could easily cross his legs and squat with both knees together.

**Figure 5:**
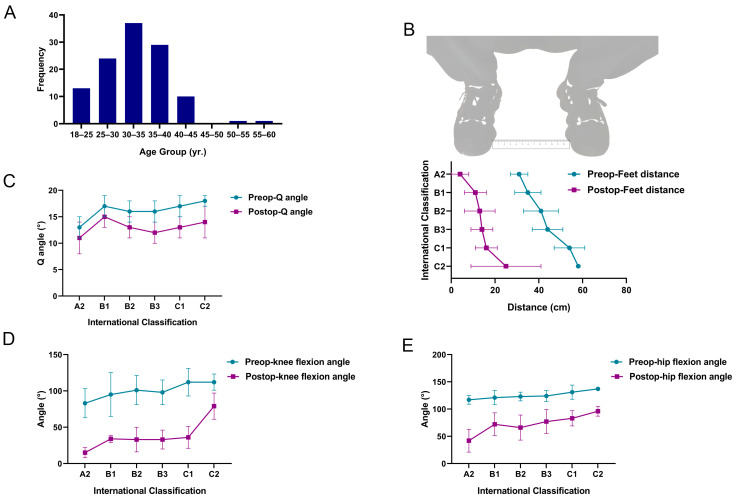
(**A**) Age distribution. (**B**) Graph showing feet distance before and after surgery. (**C**) Graph showing Q-angle before and after surgery. (**D**) Graph showing the knee flexion angle when the patient squatted maximally before and after surgery. (**E**) Graph showing the hip flexion angle when the patient squatted maximally before and after surgery. These four indicators exhibited statistically significant differences when compared before and after surgery (non-parametric test, *p* = 0.000). International classification: classification of Ye et al. [1].

All patients received arthroscopic GMC release [6]. The follow-up rate was 100% (115/115) at one month and 94.8% (109/115) at three months after surgery. In addition, because of the excellent postoperative outcome of this operation, many patients are reluctant to come to the outpatient clinic for follow-up after three months, so longer follow-up is not possible. Limited hip movement was remarkably ameliorated after surgery; patients were able to cross their legs and squat down while closing their knees together (Figure 4). In this study, most of the patients were aged 25–40 years and most were female. The benefits of arthroscopy, therefore, were clearly superior to open surgery for such patients (Figure 5A). The median Q-angle, feet distance, knee flexion angle, and hip flexion angle, were 16°, 41 cm, 104°, and 125° before surgery, and 13°, 13 cm, 31°, and 76° after surgery, respectively. Preoperative and postoperative comparisons revealed statistically significant differences (non-parametric test, *p* < 0.05). More cases were found in the B2, B3, and C1 types (Figure 5).

Measurement data of parameters related to patellofemoral instability in knee CT are presented in Table 2. As the severity of GMC increased, the values of LPD, PCA, and PTA (reflecting the degree of patellar displacement and patellar tilt) also increased (B2 to C1 of the three: 0.46 to 0.65, 19.05 to 27.25, and 19.71 to 24.26, respectively); however, this trend was not notable, and the sensitivity was poor. However, changing trends were apparent for both LPFA and PFI. LPFA gradually declined (a negative number represented inward opening) and the proportion of abnormal PFI increased, thus suggesting that the proportion of lateral displacement of the patella and patellofemoral instability increased as the degree of contracture increased.

**Figure 6:**
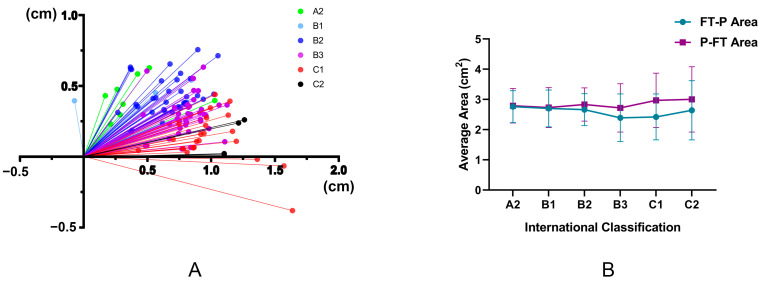
Two newly designed assessment indicators for patellofemoral instability. (**A**) With a line connecting each of the highest points of the medial and lateral femoral condyles as the *X*-axis and a line from the highest point of the lateral condyle as the *Y*-axis, it was possible to establish a coordinate system for L and α (Figure 3). This showed that as GMC severity increased, the outermost point of the patella moved outward and downward. These findings indicated that the more lateral displacement and tilt of the patella, the more serious the patellar subluxation. (**B**) Changes in P-FT area and FT-P area showed that as the severity of GMC increased, the difference between the two areas became larger (B2 to C1, 0.17 cm^2^ to 0.55 cm^2^).

The novel indicators designed in this study, distance (L) and angle (α), based on a line connecting each of the highest points of the medial and lateral femoral condyles, underwent prominent changes; the higher the degree of contracture, the longer the L, and the smaller the α (Table 2). Then, using the line connecting each of the highest points of the medial and lateral femoral condyles as an *X*-axis, and using a line from the highest point of the lateral condyle as the *Y*-axis, we established a coordinate system for L and α. This analysis showed that as the severity of GMC increased, the outermost point of the patella moved outward and downward. These findings indicated that more lateral displacement and tilt of the patella implies more serious patellar subluxation (Figure 6A). Changes in the P-FT area and the FT-P area are presented in Figure 6B; as GMC severity rose, the difference between these two areas became larger (*p* = 0.000, B2 to C1, 0.17 cm^2^ to 0.55 cm^2^) (Table 2).

As shown in Table 3, while using Ye et al.’s classification [1] as the dependent variable, we investigated the correlations between GMC severity and other relevant clinical and imaging parameters. Univariate ordered logistic regression analysis showed that a range of parameters were closely associated with GMC classification including body mass index (BMI), Preop-Q-angle, Preop-feet distance, Preop-knee flexion angle, Preop-hip flexion angle, the difference between the P-FT area and FT-P area, L, α, PTA, PFI, PCA, LPDm, and LPFA. Furthermore, multivariate analysis showed that BMI, Preop-feet distance, [P-FT area]-[FT-P area], and α were independent risk factors for the severity of GMC, in which the odds ratio (OR) or the difference in areas and Preop-feet distance was 1.074 and 1.145, respectively. This indicated that the greater these two parameters are, the more serious the possibility of GMC; α showed the opposite relationship. With respect to BMI (OR = 0.794), the smaller the weight, the higher the risk of severe GMC; this was consistent with clinical observations by our group in that most patients with severe GMC were thin.

Using [P-FT area]-[FT-P area] as the dependent variable, we investigated the associations of cross-sectional area between the patella and femur with other relevant clinical and imaging parameters of patellofemoral instability, as shown in Table 4. Univariate analysis using GLMM showed that Q-angle, feet distance, knee flexion angle, hip flexion angle, Ye et al.’s classification, L, α, PTA, PFI, PCA, LPD, and LPFA were all significantly related to the difference between areas (*p* < 0.10). Furthermore, as identified by multivariate analysis, feet distance, Ye et al.’s classification, L, PTA, PCA, and LPFA were all independent risk factors for the difference in areas. These results showed that when GMC was classified as type B3, the probability of an increase in area difference was elevated by 19.6%; when GMC was classified as type C1, this probability increased by 39.8%. Moreover, for every one-unit increase in L and PCA, the probability of an increase in area difference would be raised by 27.8% and 0.4%, respectively; an increase per unit in PTA and LPFA led to a reduction in the probability of increase in the area difference by 0.9% and 1.7%, respectively. Multivariate analysis showed that the differences in α, PFI, and LPD were not statistically significant (*p* > 0.05). This may be attributed to the condition that the change in area differences is mainly dependent on the degree of patellar displacement and patellar tilt. These three indicators often only reflect a single factor change with regard to the patella (displacement or tilt).

## 4. Discussion

In the present study, the association between GMC and patellofemoral instability was investigated from clinical physical examination and imaging data. Multivariate analysis was conducted to investigate the impact of differing severities of GMC on patellofemoral instability by combining routine assessment parameters relating to patellofemoral instability and new assessment indicators designed by our research group (feet distance, L and α, and [P-FT area]-[FT-P area]). Few studies have performed detailed multivariate analysis on the association between GMC and patellofemoral instability; there is a need for direct data validation and it is particularly easy to ignore the impact of different severities of GMC. This causes risk with regard to the clinical decision-making process for GMC patients who have knee pain or patellar subluxation. Therefore, in this study, we specifically analyzed the association between GMC and patellofemoral instability. We also analyzed the reliability of each assessment indicator for patellofemoral instability in GMC patients. This is useful when making decisions related to the clinical assessment and therapeutic regimen of GMC patients who have patellofemoral instability.

In this study, most of the GMC patients sought medical treatment due to the limitation of range of motion (ROM) in the hips that affected daily life; most patients were women (Table 1). It has been reported that GMC is more common in men than women [18]; this opposes our current findings. This may be related to the scope of the study population and the statistical methodology used. Since each patient’s cognition of this disease differs and no large-scale multi-center investigations have been performed, we cannot learn the exact gender difference in the incidence rate of GMC. However, using our own statistical data, most patients who sought medical treatment were female; it is possible that young female patients pay more attention to their posture and hip ROM. This possibility was supported by the fact that our patients were mostly aged between 25 and 35 years (Figure 5A). In addition, statistical analysis showed that BMI was significantly associated with the severity of GMC (Table 3). However, whether patients with a lower BMI are indeed prone to more severe GMC cannot be further clarified. This is because many factors can influence the severity of GMC; it is also difficult to investigate and control for these factors. Our data indicate that young women (with a lower BMI) were associated with the highest hospital visitation rate. In addition, it is also possible that males, or stronger and more obese patients, have strong and abundant lower limb muscles. Therefore, the compensatory capability of these patients would be higher and the limitation of hip ROM would not be obvious, thus leading to a lower hospital visiting rate. This possibility requires further biomechanical studies.

In addition to the limitation of hip ROM, knee pain or knee discomfort after hypermobility occurred in about one-third of the GMC patients (Table 1). Some of these patients saw a doctor due to anterior knee pain and were not diagnosed with GMC until further physical examination (probably because they had insufficient cognition of GMC in the early stage). According to some studies [6,7,8,9,18], anterior knee pain is induced by GMC and can be relieved after GMC release. Previously, our research group found that the rate of patellofemoral instability was significantly higher in GMC patients, especially those accompanied by anterior knee pain; this pain was significantly improved after arthroscopic GMC release [6]. A previous research group adopted arthroscopic GMC release combined with medial patellar retinaculum plication and lateral retinaculum release for the treatment of GMC accompanied by patellofemoral instability. These authors found that this combined form of surgery could effectively alleviate anterior knee pain caused by long-term GMC and reduced LPD when compared to arthroscopic GMC release alone [9]. The most common contracture in GMC occurs in the iliotibial tract [18]. From the anatomical perspective, contracture of the iliotibial tract and its fascia will induce LPD. Long-term patellofemoral malalignment will cause patellofemoral articular cartilage wear or osteoarthritis, thus inducing anterior knee pain or discomfort after hypermobility [19]. Therefore, it is critical to correctly assess the condition of GMC and patellofemoral instability and identify congenital patellofemoral dysplasia when selecting the therapeutic regimen for GMC patients accompanied by patellofemoral instability.

The diagnosis and conditional assessment of GMC are primarily based on clinical physical examination and intraoperative arthroscopic assessment (Figure 1 and Figure 4). The classifications of Ye et al. [1] and Zhao et al. [2] are currently the most practical and highly recognized assessment criteria. As shown in Table 1, most patients had moderate GMC; there were few patients with mild or highly severe GMC that cannot be released during surgery (classification of Ye et al. [1]: A1, A2, B1, and C2). Since the main symptom of the vast majority of GMC patients is difficulty in knee squatting, we designed a simple assessment indicator for GMC (feet distance, knee flexion angle and hip flexion angle) (Figure 1C). As shown in Table 3, feet distance was clearly associated with the severity of GMC; the larger the feet distance, the more serious the GMC (OR = 1.145). This association with the severity of GMC showed no statistically significant difference between knee flexion angle and hip flexion angle. This is probably because both waist and calf muscles are needed when the patient stands, thus weakening the ability of the two indicators to reflect the severity of GMC. However, feet distance, knee flexion angle, and hip flexion angle were all obviously ameliorated after arthroscopic GMC release (Figure 5B,D,E). Therefore, feet distance may serve as a simple assessment indicator for GMC in the clinic, although the corresponding relationship between feet distance and the severity of GMC requires further investigation.

First, it is important to assess congenital patellofemoral dysplasia (SLFR, SA, and TT-TG) for the assessment and analysis of patellofemoral instability. In this study, patients with congenital patellofemoral dysplasia were in the minority (Table 1), and there was no direct relationship with the severity of GMC (*p* > 0.05, Table 3). The Dejour classification [20,21] is commonly used for femoral trochlear dysplasia, however, this classification may be affected by subjective factors and the boundary of this classification is not obvious in some cases. In fact, SLFR, SA, and TT-TG can reflect the status of trochlear development via objective data; therefore, the Dejour classification was not adopted in this study. It has been proven in many studies that patients with trochlear dysplasia are more prone to patellofemoral instability, thus inducing patellar subluxation or dislocation [13,15,22,23]. In the present study, we did not find a significant association between the three indicators and GMC. This created favorable conditions for the analysis of GMC-induced patellofemoral instability and reduced the impact of congenital factors.

Q-angle is the most common and simple assessment indicator for the clinical assessment of patellofemoral instability and patellar subluxation [10,24]. In previous studies, we found that arthroscopic GMC release could significantly reduce the Q-angle of GMC patients, thereby improving patellofemoral instability [7]. The results of this study revealed that the preoperative Q-angle was significantly associated with the severity of GMC, but it was not an independent risk factor for the severity of GMC (*p* > 0.05, multivariate analysis, Table 3). Following arthroscopic GMC release, the Q-angle was significantly reduced; the more serious the GMC, the better the improvement (Figure 5C). Therefore, it is evident that GMC has a significant impact on patellofemoral instability and patellar subluxation. However, the Q-angle is not a reliable independent assessment indicator and may be related to the less accurate measurement method of the Q-angle.

Currently, knee imaging examinations (CT or magnetic resonance imaging) remain the most reliable method with which to assess patellofemoral instability and patellar subluxation. The most commonly used assessment indicators include PTA, PFI, PCA, LPD, and LPFA [6,11,12,14]. PTA and LPFA have higher reference values for the assessment of the degree of patellar tilt. LPD directly reflects the degree of outward displacement of the patella. The degree of patellar tilt and the degree of outward displacement both exert a significant impact on PFI and PCA; these indicators are also highly sensitive for the assessment of patellofemoral instability. Of course, outward displacement and tilt of the patella often occur simultaneously and affect each other; these changes exert an impact on the above indicators, but in different manners. In this study, univariate analysis showed that PTA, PFI, PCA, LPD, and LPFA all showed statistically significant differences when compared across GMC patients with differing severities (*p* < 0.05, Table 3), thus suggesting that GMC has a significant impact on patellofemoral instability. Moreover, the more serious the GMC, the higher the risk of patellofemoral instability; this trend is clearly evident in Table 2. The reference value for B2, B3, and C1 was high; this was due to the large sample size. However, the five indicators showed statistically significant differences in multivariate analysis, further suggesting that these indicators may have a mutual correlation or have a direct influence on each other. Of course, the influence of statistical errors (sample size, sample source, and measurement errors) cannot be excluded. When assessing patellofemoral instability, therefore, multiple indicators should be combined for analysis. It is also necessary to refer to the results of univariate analysis. A single indicator, or the blind use of multivariate models, may lead to inaccurate results.

Interestingly, the assessment indicators for GMC accompanied by patellofemoral instability that were designed by our group (the distance L and the angle α between the outermost point of the patella and the highest point of the lateral femoral condyle; [P-FT area]-[FT-P area]) were closely related to the severity of GMC (Table 3). First, the assessment of patellar subluxation in patellofemoral instability is primarily used to assess the outward displacement and tilt of the patella; L and α appear to be new and reliable assessment indicators. A previous study showed that the lateral tilt of the patella had a greater impact on anterior knee pain in the patellofemoral joint than lateral displacement [25]. Therefore, the combination of L and α is more valuable than LPD when assessing patellar subluxation. Multivariate analysis showed that α was clearly associated with the severity of GMC and served as an independent risk factor for the severity of GMC; the risk of severe GMC increased by 8.7% for each unit of decrease in α (OR = 0.913, Table 3). At the same time, as shown in Table 2 and Figure 6, the more serious the GMC, the higher the proportion of patellofemoral instability or patellar subluxation (lateral displacement and lateral tilt). Therefore, L and α can serve as simple, intuitive, and reliable assessment indicators for GMC accompanied by patellofemoral instability in the clinic.

In addition, it has been shown that the patellofemoral joint space is of importance for the soft tissue balance in the patellofemoral joint and knee ROM [26]. The outermost point of the normal patella and the highest point of the lateral femoral condyle are almost on the same sagittal plane or lie close to each other. Therefore, it can be speculated, based on our new indicators (P-FT area and FT-P area), that the difference between these two is relatively small, or may even be zero, in the normal patellofemoral joint. However, this will be significantly increased in the case of lateral tilt and lateral displacement of the patella. This area can reflect the gap between the patella and femur more comprehensively than points or lines. However, excessively small gaps or abnormal shapes (Figure 3) will worsen the patellofemoral joint wear, thus inducing osteoarthritis. Figure 6 and Table 4 show that the difference between P-FT area and FT-P area was closely related to the severity of GMC, and that severe GMC (classification of Ye et al. [1] and Preop-Feet distance) corresponded to a larger difference. Other indicators assessing lateral tilt and lateral displacement of the patella such as L, PTA, PCA, and LPFA were also closely correlated with this difference in area. Therefore, it is evident that the newly-designed indicators ([P-FT area]-[FT-P area]) can be used to verify the close association between GMC and patellofemoral instability, and may also represent reliable indicators to reflect the patellofemoral joint space and patellofemoral instability.

However, there are still some deficiencies in this study that need to be considered. First, the sample size was not large, especially for types A1, A2, B1, and C1. This was also a single-center study; therefore, our conclusions may include certain errors. Second, we used knee CT images in the extension position rather than that in the flexion position, thus leading to certain limitations when assessing the patellofemoral joint. Third, postoperative data by knee CT were not obtained; this was due to a range of factors including patient cooperation, hospitalization costs, demand for diagnosis and treatment, and the effects of CT radiation. Consequently, we were not able to investigate imaging indicators for the patellofemoral joint of GMC patients following surgery. However, a clinical physical examination (Figure 4 and Figure 5) was performed after surgery, and the Q-angle was used to indirectly assess the patellofemoral joint after surgery. In the future, our research group will address these deficiencies, expand the sample size and sources, and improve the assessment indicators to fully investigate the pathophysiological and biomechanical relationships between GMC and patellofemoral instability.

## 5. Conclusions

In this study, we combined a range of assessment indicators and performed multivariate analysis to evaluate the association between GMC classification and patellofemoral instability. We found that the more severe the GMC, the higher the risk of patellar subluxation (lateral tilt and lateral displacement). Our results also suggest that all three of the newly designed assessment indicators (feet distance, L and α, and [P-FT area]-[FT-P area]) were reliable. The former mainly reflects the severity of GMC, while the latter two are of high value for the assessment of patellofemoral instability. To conclude, our findings provide a basis and new assessment indicators for the assessment of GMC patients accompanied by patellofemoral instability. Our data may be of benefit to the decision-making process for diagnosis and treatment options.

## Figures and Tables

**Table 1:** Patient demographics (*n* = 115).

Variables		
Gender (*n*)		
Male (%)		52 (45.22)
Female (%)		63 (54.78)
Age (Mean ± SD *, year)		32 ± 6.02
BMI (Mean ± SD *)		21.75 ± 2.77
History of repeated intramuscular injections (%)		106 (92.17)
History of traumatic injuries (%)		9 (7.83)
Knee pain or discomfort (not meniscal or cruciate ligament injury)	Yes (%)	41 (35.65)
	No (%)	74 (64.35)
**Skeletal Development ^#^**		*n* (Mean ± SD *)
SLFR	≥2.5	6 (2.96 ± 0.57)
	<2.5	109 (1.64 ± 0.38)
SA	≥145°	23 (152.15 ± 5.59)
	<145°	92 (141.14 ± 2.85)
TT-TG	≥2 cm	15 (2.25 ± 0.27)
	<2 cm	100 (1.53 ± 0.35)
**Classification of GMC ^##^**		***n* (%)**
Zhao et al.	mild	14 (12.20)
	moderate	59 (51.30)
	severe	42 (36.50)
Ye et al.	A1	0 (0.00)
	A2	8 (7.00)
	B1	4 (3.50)
	B2	35 (30.40)
	B3	42 (36.50)
	C1	22 (19.10)
	C2	4 (3.50)

* SD: standard deviation. ^#^ Parameters related to skeletal development: sulcus lateral facet ratio (SLFR); sulcus angle (SA); tibial tubercle-trochlear groove distance (TT-TG). ^##^ Zhao et al.’s and Ye et al.’s classification of gluteal muscle contracture.

**Table 2:** Measurement results of patellofemoral parameters in knee CT (*n* = 115).

Classification (Mean ± SD)	[P-FT Area]-[FT-P Area] * (cm^2^)	L * (cm)	α * (°)	LPD ^#^ (cm)	PCA ^#^ (°)	PTA ^#^ (°)	LPFA ^#^ (°)	PFI ^#^ (*n*)	
								≥1.6	<1.6
A1	/	/	/	/	/	/	/	/	/
A2	0.03 ± 0.13	0.60 ± 0.26	49.95 ± 13.89	0.08 ± 0.54	10.18 ± 8.43	17.57 ± 5.60	5.45 ± 4.06	1.85 ± 0.00 (1)	1.20 ± 0.30 (7)
B1	0.03 ± 0.10	0.67 ± 0.26	53.35 ± 31.77	0.55 ± 0.44	13.26 ± 10.21	20.70 ± 6.96	5.44 ± 5.11	2.98 ± 0.00 (1)	1.41 ± 0.07 (3)
B2	0.17 ± 0.08	0.80 ± 0.20	33.74 ± 10.60	0.46 ± 0.44	19.05 ± 10.00	19.71 ± 5.27	1.29 ± 3.64	2.02 ± 0.32 (17)	1.41 ± 0.13 (18)
B3	0.34 ± 0.10	0.87 ± 0.15	18.87 ± 8.98	0.65 ± 0.44	22.84 ± 8.78	20.80 ± 6.98	1.44 ± 4.03	2.13 ± 0.42 (29)	1.37 ± 0.13 (13)
C1	0.55 ± 0.27	1.02 ± 0.28	8.33 ± 8.25	0.65 ± 0.50	27.25 ± 11.25	24.26 ± 7.79	-2.51 ± 7.75	2.18 ± 0.38 (19)	1.30 ± 0.13 (3)
C2	0.35 ± 0.11	1.18 ± 0.10	6.30 ± 5.90	1.29 ± 0.27	22.60 ± 7.41	29.58 ± 3.94	-4.52 ± 4.13	2.03 ± 0.20 (4)	/ (0)

* The measurement method is shown in Figure 3. ^#^ The measurement method is shown in Figure 2: Lateral patellar displacement (LPD); Patellar congruency angle (PCA); Patellar tilt angle (PTA); Lateral patellofemoral angle (LPFA, “-” number means angle opening inward); Patellofemoral index (PFI).

**Table 3:** Factors associated with international classification of gluteal muscle contracture (Ye et al.) in the ordinal logistic regression analysis.

			Univariate Analysis			Multivariate Analysis		
Factors			OR	95% CI	*p*	OR	95% CI	*p*
Clinical findings	Body mass index	(kg/m^2^)	0.849	0.750–0.961	0.010 *	0.794	0.666–0.945	0.009 ^#^
	Age Group	(By 5 years)						
		18~	0.733	0.018–29.312	0.869			
		25~	1.073	0.028–40.407	0.970			
		30~	0.501	0.014–18.430	0.707			
		35~	0.616	0.017–22.920	0.793			
		40~	0.491	0.012–20.471	0.709			
		45~	/	/	/			
		50~	1.000	0.007–152.627	1.000			
		55~	-	-	-			
	Gender	(Male/Female)	0.811	0.416–1.576	0.536			
	Preop-Q angle	(°)	1.319	1.121–1.553	0.001 *	1.082	0.856–1.369	0.511
	Preop-Feet distance	(cm)	1.195	1.139–1.255	0.000 *	1.145	1.062–1.234	0.000 ^#^
	Preop-Knee flexion angle	(°)	1.028	1.010–1.047	0.002 *	0.977	0.946–1.009	0.159
	Preop-Hip flexion angle	(°)	1.068	1.033–1.104	0.000*	1.052	0.996–1.112	0.071
CT findings	[P-FT area]-[FT-P area]	(mm^2^)	1.103	1.075–1.133	0.000 *	1.074	1.036–1.114	0.000 ^#^
	L	(cm)	130.974	24.928–687.457	0.000 *	1.141	0.099–13.171	0.916
	α	(°)	0.864	0.833–0.896	0.000 *	0.913	0.866–0.964	0.001 ^#^
	Patellar tilt angle	(°)	1.090	1.036–1.147	0.001 *	0.991	0.879–1.119	0.887
	Patellofemoral index	(<1.6/≥1.6)	0.176	0.082–0.374	0.000 *	0.489	0.185–1.288	0.148
	Patellar congruency angle	(°)	1.079	1.043–1.117	0.000 *	0.963	0.913–1.014	0.153
	Lateral patellar displacement	(cm)	4.055	1.968–8.365	0.000 *	3.139	0.838–11.775	0.090
	Lateral patellofemoral angle	(°)	0.868	0.811–0.930	0.000 *	1.096	0.951–1.265	0.205
	Sulcus lateral facet ratio		1.005	0.507–1.992	0.989			
	Sulcus angle	(<145°/≥145°)	0.533	0.231–1.229	0.140			
	Tibial tubercle-trochlear groove distance	(<2 cm/≥2 cm)	0.934	0.350–2.497	0.892			

* Risk factors with *p* values < 0.10 in univariate analysis were included in a multivariate analysis. ^#^ Independent risk factors (*p* < 0.05) was identified by a multivariate logistic regression analysis. -Reference; /None.

**Table 4:** Factors associated with the difference between P-FT area and FT-P area in the generalized linear mixed model.

			Univariate Analysis			Multivariate Analysis		
Factors			OR	95% CI	*p*	OR	95% CI	*p*
Clinical findings	Body mass index	(kg/m^2^)	0.991	0.976–1.005	0.206			
	Age Group	(By 5 years)						
		18~	-	-	-			
		25~	1.057	0.912–1.223	0.461			
		30~	0.998	0.870–1.145	0.982			
		35~	1.077	0.934–1.241	0.303			
		40~	1.169	0.977–1.398	0.086			
		45~	/	/	/			
		50~	1.270	0.817–1.974	0.285			
		55~	0.979	0.630–1.522	0.926			
	Gender	(Male/Female)	0.982	0.907–1.064	0.660			
	Preop-Q angle	(°)	1.020	1.002–1.040	0.030 *	1.003	0.990–1.017	0.618
	Preop-Feet distance	(cm)	1.009	1.006–1.013	0.000 *	0.996	0.992–1.000	0.033^#^
	Preop-Knee flexion angle	(°)	1.002	1.000–1.004	0.057 *	1.001	0.999–1.002	0.392
	Preop-Hip flexion angle	(°)	1.004	1.000–1.007	0.054 *	0.997	0.994–1.000	0.051
	Ye et al.’s classification	(gluteal muscle contracture)			*			
		A2	-	-	-	-	-	-
		B1	1.004	0.840–1.198	0.967	1.050	0.894–1.234	0.546
		B2	1.148	1.025–1.287	0.017	1.031	0.924–1.151	0.578
		B3	1.355	1.213–1.516	0.000	1.196	1.057–1.353	0.005 ^#^
		C1	1.687	1.496–1.900	0.000	1.398	1.204–1.623	0.000 ^#^
		C2	1.379	1.155–1.647	0.000	1.212	0.991–1.481	0.061
CT findings	L	(cm)	1.690	1.474–1.937	0.000 *	1.278	1.121–1.458	0.000 ^#^
	α	(°)	0.992	0.990–0.994	0.000 *	0.998	0.995–1.001	0.183
	Patellar tilt angle	(°)	1.008	1.002–1.013	0.009 *	0.991	0.985–0.998	0.008 ^#^
	Patellofemoral index	(<1.6/≥1.6)	1.169	1.082–1.262	0.000 *	1.011	0.959–1.066	0.686
	Patellar congruency angle	(°)	1.010	1.006–1.013	0.000 *	1.004	1.001–1.007	0.007 ^#^
	Lateral patellar displacement	(cm)	1.127	1.042–1.219	0.003 *	0.940	0.872–1.014	0.107
	Lateral patellofemoral angle	(°)	0.982	0.975–0.988	0.000 *	0.983	0.976–0.991	0.000 ^#^
	Sulcus lateral facet ratio		0.965	0.889–1.047	0.389			
	Sulcus angle	(<145°/≥145°)	1.038	0.939–1.146	0.466			
	Tibial tubercle-trochlear groove distance	(<2 cm/≥2 cm)	1.029	0.914–1.158	0.633			

* Risk factors with *p* values <  0.10 in univariate analysis were included in a multivariate analysis. ^#^ Independent risk factors (*p* < 0.05) was identified by a multivariate generalized linear mixed model. -Reference; /None.

## Data Availability

The raw data are available upon reasonable request from the corresponding author (B.C.).
